# Lesser relevance markers in Chinese academic spoken English corpus: a cross-disciplinary study on pragmatic features

**DOI:** 10.3389/fpsyg.2023.1297038

**Published:** 2023-11-27

**Authors:** Yang Yu, Gang Zeng

**Affiliations:** School of Foreign Languages, Dalian Maritime University, Dalian, China

**Keywords:** lesser relevance markers, academic spoken English lectures, Chinese scholars, cross-cultural communication, cross-disciplinary study

## Abstract

This study explores the usage characteristics and pragmatic functions of lesser relevance markers in academic spoken English lectures presented by Chinese scholars. A qualitative and quantitative approach is employed using the Chinese Academic Spoken English Corpus (CASEC), which encompasses disciplines in science, engineering, humanities, and social sciences. The findings reveal that Chinese scholars use lesser relevance markers less frequently compared to native English speakers. These differences in usage highlight the influence of language background, disciplinary culture, and communication conventions on the realization of informing evaluation, topic handling, and interactivity. Furthermore, within the Chinese scholars’ group, humanities and social sciences scholars tend to use lesser relevance markers more frequently than science and engineering scholars. This research enhances our understanding of the multifaceted pragmatic roles of lesser relevance markers and offers insights into cross-cultural academic communication and English teaching.

## 1 Introduction

Lesser relevance markers, which constitute a substantial subgroup of discourse markers (Caffi, 1999; Deroey K. and Taverniers, 2012; Deroey, 2015), perform a crucial role in language by explicitly indicating the subordinate or secondary relevance of expressed content. These markers, classified as metadiscourse, fulfill various pragmatic functions, including conveying the speaker’s intent, expressing emotions and attitudes, and emphasizing the secondary nature of specific information (Zare, 2020, 2023; Zare et al., 2022). At the discourse level, they enhance the speaker’s oral expression and promote audience comprehension (Deroey, 2011, 2015). Consequently, lesser relevance markers are not only a fundamental aspect of academic written language but also a pivotal criterion for evaluating academic spoken language (Hunston, 1994, 2000). Previous research has highlighted significant variations in the usage frequency of lesser relevance markers across disciplines, with reduced usage potentially impacting readers’ comprehension and evaluation of academic papers (Hyland, 1998, 2004, 2005; Xiao, 2010). Furthermore, Wang and Sun (2018) found that factors such as speaker gender, discourse mode, and academic discipline influence the use of lesser relevance markers in academic spoken language, while Yankova and Vassileva (2021) observed that Bulgarian scholars tend to use lesser relevance markers less frequently compared to native English speakers, potentially attributable to cultural backgrounds. Hence, the study of lesser relevance marker distribution across disciplines and cultures is essential for understanding their pragmatic and discourse functions in diverse academic contexts and cultural settings.

Although current research on the pragmatic functions of lesser relevance markers mainly focuses on everyday communication and academic discourse, in everyday communication, markers such as “anyway” (Takahara, 1998) and, in academic discourse, phrases such as “Let me introduce” (Ädel, 2012) serve evaluative functions. In everyday language, terms such as “may” (Jucker et al., 2003) and, in academic discourse, modifiers such as “a little bit” (Mauranen, 2004) and “big” (Lin, 2010) have topic management functions. Additionally, in academic discourse, “actually” serves an information evaluation function (Caffi, 1999; Pichler, 2007; Bolden, 2009). There are two notable gaps in the existing literature. First, the majority of studies concentrate on academic written language (Fraser, 1980, 1999, 2009), with limited attention given to academic spoken genres, such as lectures (Thompson, 2003; Lee and Subtirelu, 2015). Second, research primarily centers on native English speakers (Lemke, 1998; Pichler, 2007; Neely and Cortes, 2009; Pan, 2011), with limited exploration of non-native English speakers’ utilization of lesser relevance markers, particularly in terms of Chinese scholars’ cross-disciplinary usage in academic spoken English.

In light of these gaps, this study addresses the need for more comprehensive research by constructing a Chinese Academic Spoken English Corpus and employing qualitative and quantitative research methods. The primary objective is to investigate the cross-disciplinary usage features of lesser relevance markers in Chinese scholars’ academic spoken English, thereby uncovering differences in metalinguistic functions. The findings from this study will facilitate a deeper understanding of the mechanisms and patterns in cross-disciplinary academic spoken English among Chinese scholars and provide valuable insights for the teaching of academic spoken English.

## 2 Functions and cultural influences on discourse markers

In our endeavor to comprehend the nuances of spoken academic English, particularly in a cross-cultural setting, an in-depth examination of discourse markers is indispensable. One cannot explore this terrain without acknowledging the seminal work of Schiffrin (1987), who has elucidated the multifaceted functions of discourse markers. These functions range from managing relevance and signaling topic shifts to structuring ideas and organizing spoken interaction.

Recent literature further enriches this area of study. For instance, Aijmer (2022) investigated the evolution of the English adverb “basically” in two comparable British spoken language corpora. Her study highlighted its transition from a core meaning to a pragmatic marker with a mitigating function. Such markers, especially in their developmental phases, offer intriguing insights into language evolution and the effects of sociocultural shifts over relatively short periods. Kashiha (2022), on the other hand, broadens the lens by comparing the use of metadiscourse markers in academic and political speeches. Through this comparison, the study emphasizes the salient differences in audience engagement and linguistic choices based on discourse type, underscoring the importance of audience awareness in spoken interactions. Tantucci and Wang (2022), in contrast, explored conversations in Mandarin Chinese and American English, establishing a clear correlation between discourse markers and alignment. Their findings not only emphasize the pivotal role of alignment in interactive dialogs but also suggest potential avenues for technological advancements, particularly in the realm of human–machine interactions. A fresh perspective emerges from Adel’s work in 2023 (Adel, 2023), which promotes a “fluidity” approach in metadiscourse research. Rather than a rigid word-level “marker” approach, Adel’s methodology calls for context-based discourse function analysis. This approach resonates particularly when considering student speeches, a domain that remains relatively underexplored despite its ubiquity. Lastly, Huang et al. (2023) delve into the intricate relationship between second language fluency and the deployment of discourse markers. The study presents a compelling case for understanding the developmental trajectory of discourse markers, such as “well” and “you know,” as language proficiency evolves, emphasizing the crucial role immersive experiences play in shaping this journey.

Notably, within the vast spectrum of discourse markers, lesser relevance markers carve out a niche for themselves by explicitly denoting information of comparatively lower relevance. It is pertinent to mention here that the choice and deployment of lesser relevance markers are influenced not only by linguistic factors but also by cultural inclinations and disciplinary conventions. This research, while acknowledging these influences, aims primarily to spotlight cross-cultural variations, seeking to fathom how different cultural backgrounds may mold linguistic choices in academic spoken English.

## 3 Pragmatic functional classification of lesser relevance markers

In academic lectures, lesser relevance markers are commonly regarded as tools used to embody interpersonal functions (Ädel, 2012). Speakers utilize these markers to inform the audience about varying degrees of relevance, highlighting information that is of lesser importance. This helps the audience better understand and interpret the intended meaning of the discourse. Furthermore, lesser relevance markers serve pragmatic functions such as indicating the desire to end a topic, yielding the floor, or mitigating potential face threats. Consequently, marking the pragmatic appropriateness of lesser relevance is crucial, particularly in high-risk genres such as lectures (Kiewra, 2002; Titsworth and Kiewra, 2004). Speakers can only assist listeners in fully understanding, absorbing, and retaining the key points of the discourse, thereby achieving comprehension and successfully conveying the lecture, by appropriately using lesser relevance markers to distinguish between relevant and lesser relevant information.

Capone’s exploration of “Pragmemes” (Capone, 2005), specifically in relation to English and Italian, significantly contributes to our understanding of how linguistic structures and meaning-making processes differ cross-culturally. His comprehensive analysis provides valuable insights into these cross-cultural and cross-linguistic variations. Building on Capone’s work, Fetzer’s book “Pragmemes in Discourse” (Fetzer, 2016) delves deeper into the role of pragmemes in structuring and organizing discourse, adding another layer to our comprehension. Based on the British Academic Spoken English (BASE) corpus, Deroey K. and Taverniers (2012) consider lesser relevance markers as tools for interpersonal communication, used to identify less relevant viewpoints or information. They propose a pragmatic functional classification for these markers, suggesting that their functions overlap (Deroey, 2011). Jock Wong’s work, “The Culture of Language” (Wong, 2016), brings another dimension to the conversation by exploring the intricate relationship between language and culture. Wong’s investigations shed light on the profound influence of cultural norms, beliefs, and values on language usage and choice, reinforcing the notion that while linguistic structures may be universal, their usage is deeply rooted in cultural nuances.

While functional considerations related to the subject matter may dictate the use of these markers, cultural differences often play a pivotal role in linguistic choices. Various cultures emphasize different communicative norms and values, which can lead to preferences for certain discourse markers over others. Even within a single discipline, speakers from different regions with diverse cultural backgrounds may structure their discourse differently, selecting particular markers to emphasize, transition, or mitigate. Based on these theoretical foundations and by combining their pragmatic functional classifications, we propose the following five categories for the pragmatic functions of lesser relevance markers: informing evaluation, topic handling, lecturer strategy, appraisal, and interactivity. The specifics of each category are explained as follows:

(1) Informing evaluation: This pragmatic function involves directly evaluating the lesser relevance or irrelevance of information in the lecture, for example, using metalinguistic nouns (Deroey K. L. B. and Taverniers, 2012) that indicate unimportance or irrelevance, such as “irrelevant” and “unimportant,” or employing markers that signal a shift from the main topic to secondary content, such as “anyway,” “aside,” and “by the way”. Examples are as follows:

In the sentence “This point, though not entirely irrelevant, is not central to our current discussion.”, the lesser relevance marker “irrelevant” expresses that although the viewpoint is not completely irrelevant, it is not the core of the current discussion. The use of such lesser relevance markers allows readers or listeners to clearly identify the speaker’s stance and better understand the overall direction and emphasis of the topic.

In the sentence “That detail is unimportant for our current understanding of the concept.”, the lesser relevance marker “unimportant” communicates that this detail is not significant for comprehending the concept. Employing this lesser relevance marker helps emphasize the main viewpoints or concepts by de-emphasizing or excluding certain minor details, allowing the audience to focus on the more critical aspects.

In the sentence “Anyway, let us get back to the main topic.”, the lesser relevance marker “anyway” indicates that the following dialog will return to the main topic. This marker aids in transitioning between topics, guiding the audience’s attention from potential digressions or trivial details back to the core of the subject, thereby maintaining the coherence and consistency of the discourse.

In the sentence “Let me put what we just covered aside.”, the lesser relevance marker “aside” signals the suspension of the previous dialog. By implying that the aforementioned content will be temporarily set aside, this marker paves the way for new topics or directions, adjusting the rhythm and direction of the discourse. This not only maintains the fluency of the discourse but also allows for flexible adjustments and guidance of the dialog’s progression according to the discussion’s requirements.

(2) Topic handling: This pragmatic function primarily concerns how the lecturer addresses new topics, rather than directly commenting on the relevance or irrelevance of information. It can be achieved by using metalinguistic nouns that indicate limited time or space, such as “briefly” or “quickly.” Examples are as follows:

In the sentence “I’ll just briefly explain this concept.”, the lesser relevance marker “briefly” indicates that the concept will be concisely explained. Employing such linguistic means in academic lectures or presentations helps highlight the hierarchy and priorities of information. Emphasizing “briefly” enables lecturers to better manage time, enabling the audience to focus on key, and potentially more complex, issues. It also indicates that while the concept possesses some importance, it is not central to the main topic; thus, a simple introduction suffices.

In the sentence “I’ll quickly go over the history of this theory.”, the lesser relevance marker “quickly” indicates that the history of the theory will be reviewed swiftly. This linguistic expression reflects the lecturer’s flexibility in time allocation and precise grasp of the subject’s core aspects. Using “quickly” emphasizes the lecturer’s intent to focus the audience’s attention more on the theory’s current applications or other critical facets, rather than delving into historical details. Additionally, by emphasizing “quickly,” lecturers can effectively cover more content within a limited time, ensuring overall coherence and completeness.

(3) Lecturer strategy: This pragmatic function mainly focuses on how the lecturer describes the completeness of lecture information, rather than evaluating the information itself. Common metalinguistic nouns include “forget” or “not know.” Examples are as follows:

In the sentence “I forgot to mention earlier that this equation has a unique property.”, the lesser relevance marker “forget” demonstrates a self-correcting strategy, wherein previously omitted information is introduced in the lecture. Utilizing this expression emphasizes the importance of the equation’s unique property and highlights the common phenomenon in human lectures where people sometimes forget some information in a natural, informal way. By acknowledging this, the lecturer establishes rapport with the audience while underscoring the significance of the omitted information.

In the sentence “I do not know the specific details of this process.”, the lesser relevance marker “not know” expresses an honest and transparent attitude. The lecturer acknowledges not knowing the specific details of the process, establishing their credibility by demonstrating a clear understanding of the scope of their knowledge and an honest admission. In academic settings, such candid acknowledgment promotes openness and critical thinking, encouraging the audience to independently seek information or ask further questions. Simultaneously, it ensures the accuracy of lecture content, avoiding misleading uncertain information.

(4) Appraisal: This pragmatic function clearly indicates that certain information in the lecture is unimportant for subsequent evaluation. It relies predominantly on external evaluation requirements rather than the lecturer’s personal judgment of the information’s importance. Common metalinguistic nouns include “not examine” or “not learn.” Examples are as follows:

In the sentence “The specifics of this experiment will not be examined in your final assessment.”, the lesser relevance marker “not examine” clearly indicates that specific details of the experiment will not be tested in the final evaluation. This statement establishes the boundaries of the assessment and guides the audience’s attention toward other important aspects of the subject content. In teaching contexts, clearly communicating to students what content will and will not be assessed is crucial as it enables students to rationally allocate their learning time and energy and focus on mastering core concepts and skills. Additionally, it demonstrates respect for the audience by providing them with clear expectations and preparation for the upcoming evaluation.

In the sentence “Learning these equations by heart is not required for this course.”, the lesser relevance marker “not learn” informs the audience that memorizing these equations through rote learning is unnecessary. This statement reflects the course designers’ understanding and values regarding the learning process. Many teaching philosophies prioritize understanding and applying knowledge over mechanical memorization. By explicitly expressing this point, the lecturer encourages the audience to pursue deeper understanding rather than shallow memorization. This helps create a learning environment that promotes exploratory thinking and critical analysis.

(5) Interactivity: This pragmatic function primarily focuses on controlling the delivery of information in the lecture and providing the audience with specific operational guidance on how to pay attention and take notes. Common metalinguistic nouns include “ignore” or “never mind.” Examples are as follows:

In the sentence “You can ignore the complex calculations here. The concept is what matters.”, the lesser relevance marker “ignore” gives directive guidance that the complex calculations can be disregarded in the current learning environment, with the focus on grasping the core concepts. This expression emphasizes the priority of understanding concepts over pure calculation skills. Particularly in teaching abstract theoretical courses, lecturers may avoid delving into complex calculations to guide students’ understanding and grasp of core viewpoints. By using “ignore,” the lecturer consciously redirects students’ attention from the specific calculation process to comprehending the theoretical framework and conceptual structure.

In the sentence “Never mind taking notes on this section, I’ll provide a handout later.”, the lesser relevance marker “never mind” informs the audience that they need not take notes on this section as handouts will be provided later. This statement showcases the lecturer’s care and support for the students’ learning process, ensuring that students can fully concentrate on listening and understanding without the burden of note-taking. Additionally, it highlights the section’s special nature, possibly containing complex charts or detailed data that is better conveyed through written materials. This improves teaching efficiency, ensures the accuracy of information, and encourages students to actively participate in class.

In summary, the informing evaluation function assesses the importance of information, while the topic handling function demonstrates the way topics are addressed. The lecturer strategy function showcases the lecturer’s approach to ensuring that the content is comprehensive. The appraisal function emphasizes the significance of certain information in subsequent assessments, and the interactivity function directs the audience’s attention and note-taking. These markers serve both pragmatic and interpersonal purposes. The appropriate use of these markers is crucial in high-risk genres such as lectures as it contributes to the audience’s comprehension and retention of key information. Moreover, examining cross-cultural and cross-linguistic variations in the utilization of these markers provides valuable insights into the differences in linguistic structures and processes of meaning-making across cultures. Additionally, the role of cultural norms and values in shaping linguistic choices cannot be disregarded. While the disciplinary subject matter may influence the functional use of these markers, the research primarily aims to clarify how cultural nuances intertwine with these disciplinary differences. Gaining an understanding of these functions enables a comprehensive understanding of the logical language and organizational structure of cross-disciplinary academic lectures.

## 4 Research methodology

### 4.1 Research questions

This study primarily aims to investigate the following research questions:

1. What are the cross-disciplinary usage frequencies of lesser relevance markers in academic lectures delivered by Chinese scholars compared to those given by English native scholars?
2. What are the cross-disciplinary pragmatic functional characteristics of lesser relevance markers in academic lectures delivered by Chinese scholars compared to those delivered by English native scholars?
3. What are the cross-disciplinary pragmatic functional characteristics of lesser relevance markers within the disciplines of Chinese science and engineering scholars and humanities and social sciences scholars?

### 4.2 Research method overview

The data for this study are derived from two main sources: the Chinese Academic Spoken English Corpus (CASEC) and the Michigan Corpus of Academic Spoken English (MICASE). In CASEC, the lectures encompass various formats and have undergone meticulous sentence-by-sentence checking to ensure accuracy. Detailed metadata tagging has also been carried out to provide additional information about the lectures. MICASE, on the other hand, is a reference corpus. As for the analysis techniques, the data analysis process follows a series of steps. It starts with preliminary screening to identify lesser relevance markers. Then, corpus retrieval is performed to retrieve examples containing the candidate words from both CASEC and MICASE using a corpus processing tool called Sketch Engine. Pragmatic function judgments are made independently by researchers to determine the specific pragmatic functions of the candidate words in the retrieved examples. In the classification and statistics stages, the candidate words are classified based on the results of the pragmatic function judgments, and statistical analyses are performed. Comparative analysis is then conducted to compare the classification results between CASEC and MICASE. Finally, in the in-depth interpretation stage, concordancing and further pragmatic analyses are conducted for lesser relevance markers that exhibit significant differences in pragmatic functions, considering China’s cultural background and differences between disciplines.

### 4.3 Corpus collection and screening

This study utilizes a corpus of academic spoken lectures, a prominent genre, covering two major disciplinary fields: science and engineering and humanities and social sciences. Both sections of the corpus are sufficiently large, with 209 lectures and 532,601 words in the science and engineering section and 83 lectures and 305,560 words in the humanities and social sciences section. The lectures include various formats such as large lectures, small group discussions, academic conferences, and doctoral dissertation defenses. Every transcript in the corpus has been meticulously checked sentence-by-sentence to ensure accuracy. Furthermore, detailed metadata tagging has been carried out, including information about the background of the lectures, discipline classification, lecture level, interactivity, audience size, lecturer gender, and other relevant metadata. Overall, this research corpus is constructed with the principles of representativeness, broad coverage, sufficient samples, and accurate tagging to meet the requirements for the study of spoken academic corpora. It is officially named the Chinese Academic Spoken English Corpus (CASEC), which is further divided into the science and engineering section (CASEC-T) and the humanities and social sciences section (CASEC-HS).

The MICASE corpus, an academic spoken corpus created and maintained by the University of Michigan from 1997 to 2001 for teaching and research purposes, is selected as a reference corpus. It has a total vocabulary of approximately 1.8 million words and covers multiple disciplines including physics, engineering, biology, medicine, social sciences, education, humanities, and arts. The corpus consists of a total of 152 discourse events. Additionally, all spoken transcripts are extensively annotated, including information about discourse mode, speaker identity, gender, discipline, and non-verbal cues. Specific details of the corpus are provided in Table 1.

**Table 1:** Overview of CASEC and MICASE corpora.

Corpus	Academic classification	Number of lectures	Word count	Total duration
CASEC	Science and Engineering (CASEC-T)	209	532,601	Approximately 61 h
	Humanities and Social Sciences (CASEC-HS)	83	305,560	Approximately 47 h
	Total	292	838,161	Approximately 108 h
MICASE	Science and Engineering (MICASE-T)	68	684,232	–
	Humanities and Social Sciences (MICASE-HS)	71	855,016	–
	Total	139	1,539,248	Approximately 190 h

### 4.4 Data analysis process

The data analysis process of this study follows a series of steps: preliminary screening, corpus retrieval, judgment of pragmatic functions, classification and statistics, comparative analysis, and in-depth interpretation.

At the preliminary screening stage, a candidate list of lesser relevance markers required for this study is constructed based on the classification by Deroey K. and Taverniers (2012). This forms the foundation for the subsequent corpus retrieval work.

Next, candidate items proposed in the preliminary analysis stage are comprehensively retrieved in the prebuilt CASEC corpus and the MICASE reference corpus using Sketch Engine, a corpus processing tool. This retrieval yields example sentences containing the candidate words.

In the following stage of pragmatic function judgment, researchers independently determine the specific pragmatic function of the candidate words in the example sentences. It is important to note that researchers must strictly distinguish between the core semantic function and the contextual pragmatic function of words during the judgment process. These two functions should not be equated simplistically. To accurately identify the actual pragmatic function of a word, researchers need to thoroughly examine contextual cues such as collocation with other markers, syntactic structure, and contextual content.

In the classification and statistics stages, researchers classify the candidate words based on the results of pragmatic function judgments and perform statistical analyses.

Subsequently, in the comparative analysis stage, the classification results of the candidate words in the CASEC corpus and the MICASE reference corpus are compared statistically to identify significant differences between them.

Finally, in the in-depth interpretation stage, for lesser relevance markers that exhibit significant differences in pragmatic functions, in-depth pragmatic analyses are conducted based on China’s cultural background and the differences between disciplines. First, at the preliminary screening stage, based on Deroey K. and Taverniers' (2012) classification of lesser relevance markers, a candidate list of lesser relevance markers required for this study is constructed, laying the foundation for the subsequent corpus retrieval work, where concordancing and pragmatic functional analysis will be applied.

## 5 Analysis and discussion

### 5.1 Overall cross-disciplinary usage frequency of lesser relevance markers in Chinese scholars’ academic spoken English

This study aims to conduct a comprehensive analysis of the overall usage frequency of lesser relevance markers in Chinese scholars’ academic spoken English. To achieve this, the study utilized a pragmatic functional classification system and extracted five main categories of lesser relevance markers from the CASEC and MICASE corpora. The research involved preliminary screening, corpus retrieval, determination of pragmatic function, categorization, and comparative statistical analysis. Previous studies, such as Yankova and Vassileva (2021), have indicated substantial variations in the usage frequency of lesser relevance markers across different cultural backgrounds. Building on these findings, this study delves deeper into the distinctions between science and engineering and humanities and social sciences disciplines to investigate whether variations exist in the use of these markers across different cultures and disciplines. The overall usage frequency of lesser relevance markers in academic English lectures by Chinese scholars in science and engineering can be found in Table 2.

**Table 2:** Cross-disciplinary comparison of the overall usage frequency of lesser relevance markers in CASEC-T and MICASE-T.

	CASEC-T frequency	MICASE-T frequency	CASEC-T normalized frequency	MICASE-T normalized frequency	Chi-square value (*χ*^2^)	Significance level (*p*)
Overall usage frequency of lesser relevance markers in science and engineering	4,293	6,734	8,062	9,842	−105.62	0.000

Table 3 compares the overall usage frequencies of lesser relevance markers by Chinese scholars in the fields of humanities and social sciences in academic spoken English lectures.

**Table 3:** Cross-disciplinary comparison of the overall usage frequency of lesser relevance markers in CASEC-HS and MICASE-HS.

	CASEC-HS frequency	MICASE-HS frequency	CASEC-HS normalized frequency	MICASE-HS normalized frequency	Chi-square value (*χ*^2^)	Significance level (*p*)
Overall usage frequency of lesser relevance markers in humanities and social sciences scholars	2,895	10,117	9,474	11,833	−112.70	0.000

According to Hyland (1998) and Hyland and Tse’s research results (Hyland, 2004), the use of these markers varies significantly across disciplines. When academic papers use fewer lesser relevance markers, it can affect readers’ comprehension and evaluation of the papers in that specific discipline. In academic spoken English lectures, these markers play a vital role in conveying viewpoints, supporting arguments, and organizing lecture structures. By using them appropriately, lecturers can cite previous research results, provide evidence to support their own viewpoints, and help the audience better understand the content of the lecture. However, a reduced use of these markers may lead to the audience inaccurately understanding the lecturer’s viewpoints and arguments, questioning the credibility and academic nature of the lecture. Therefore, studying the distribution of these markers’ usage across disciplines and cultures is crucial for understanding their pragmatic and discourse functions in various academic contexts and cultural settings.

Table 2 presents data on the overall frequency of lesser relevance markers used by Chinese scholars in the fields of science and engineering in academic spoken English lectures. According to the results of the chi-square test (*χ*^2^ = −105.62, *p* = 0.000), the usage frequency of these markers by Chinese science and engineering scholars is significantly lower than that of English native speakers. Similarly, Table 3 shows the overall frequency of lesser relevance markers used by Chinese humanities and social sciences scholars in academic spoken English lectures. According to the results of the chi-square test (*χ*^2^ = −112.70, *p* = 0.000), the usage frequency of these markers by Chinese humanities and social sciences scholars is also significantly lower than that of English native speakers.

The findings reveal that Chinese scholars in the fields of science and engineering, as well as humanities and social sciences, use these markers significantly less frequently compared to English native speakers. This discrepancy may be influenced by factors such as language background, disciplinary culture, and academic communication conventions (Shaw et al., 2010; Zare, 2020, 2023). To enhance the quality of lectures and ensure accurate delivery of content, Chinese scholars should focus on increasing their use of these markers, thereby improving the understandability and academic nature of their lectures.

### 5.2 Cross-disciplinary pragmatic functional classification and comparison of lesser relevance markers in Chinese scholars’ academic spoken English in science and engineering

For an in-depth analysis, we retrieved five major types of pragmatic functional lesser relevance markers from the CASEC and MICASE corpora, based on Deroey K. and Taverniers' (2012) classification. Types with frequencies of 0 and standardized frequencies below 5 in both corpora were excluded. Given the disciplinary differences between science and engineering and humanities and social sciences, to further understand whether there are variations in the pragmatic function usage of lesser relevance markers across different disciplines, the overall distribution of the pragmatic function of these markers in science and engineering can be seen in Table 4.

**Table 4:** Comparison of usage frequencies of five pragmatic functions of lesser relevance markers in CASEC-T and MICASE-T.

Five pragmatic functions of lesser relevance markers		CASEC-T frequency	MICASE-T frequency	CASEC-T normalized frequency	MICASE-T normalized frequency	Chi-square value (*χ*^2^)	Significance level (*p*)
1	Informing evaluation	108	229	203	335	−1.38	0.240
2	Topic handling	337	619	633	905	0.00	0.975
3	Lecturer strategy	11	29	21	42	−0.74	0.389
4	Appraisal	4	20	8	29	−2.86	0.091
5	Interactivity	3,833	5,837	7,197	8,531	81.52	0.000
	Total	4,293	6,734	8,062	9,842	-	-

The results show that Chinese scholars in science and engineering use three categories of markers less frequently than native English speakers, namely Informing Evaluation, Lecturer Strategy, and Appraisal. However, the category of Interactivity (*χ*^2^ = 81.52, *p* = 0.000) stands out as the most frequently used pragmatic function of lesser relevance markers by Chinese scholars in science and engineering. This finding suggests that there are cross-cultural distinctions in the usage of these markers, emphasizing the importance of understanding their implications in different disciplinary contexts.

To more accurately identify specific significant lesser relevance markers within the overall distributions of Interactivity, which have a significant difference, we meticulously counted the frequency of each marker in the two corpora. After normalization and paired chi-square tests, the frequencies of lesser relevance markers with significant differences are shown in Table 5.

**Table 5:** Metalinguistic nouns of lesser relevance markers with significant differences in CASEC-T and MICASE-T.

Pragmatic functions of lesser relevance markers with significant difference		CASEC-T frequency	MICASE-T frequency	CASEC-T normalized frequency	MICASE-T normalized frequency	Chi-square value (*χ*^2^)	Significance level (*p*)
*Metalinguistic noun of interactivity*							
1	About/With	2,593	8,880	8,486	10,386	−82.81	0.0000

From Table 5, the analysis compared the frequency of specific lesser relevance markers in two corpora, revealing significant differences. One such marker, “about/with,” was found to have lower frequencies in the CASEC-T corpus compared to MICASE-T, with negative chi-square values. It was observed that native English speakers in the science and engineering field frequently use these prepositions in their academic lectures, signifying their function in introducing topics, connecting concepts, and expressing relationships. However, Chinese scholars in the same field use these prepositions infrequently, possibly due to unfamiliarity or oversight during the English learning process (Sinclair, 2004). This discrepancy in language usage may limit the performance of Chinese scholars on the international academic stage as these expressions are considered foundational for effective communication.

### 5.3 Cross-disciplinary pragmatic functional classification and comparison of lesser relevance markers in Chinese scholars’ academic spoken English in humanities and social sciences

The overall distribution of the pragmatic function of lesser relevance markers in humanities and social sciences is presented in Table 6.

**Table 6:** Comparison of usage frequencies of five pragmatic functions of lesser relevance markers in CASEC-HS and MICASE-HS.

Five pragmatic functions of lesser relevance markers		CASEC-HS frequency	MICASE-HS frequency	CASEC-HS normalized frequency	MICASE-HS normalized frequency	Chi-square value (*χ*^2^)	Significance level (*p*)
1	Informing evaluation	63	418	206	489	−102.51	0.000
2	Topic handling	224	731	733	855	−57.76	0.000
3	Lecturer strategy	8	28	26	33	−2.14	0.144
4	Appraisal	11	19	36	22	0.00	0.977
5	Interactivity	2,589	8,921	8,473	10,434	−824.67	0.000
	Total	2,895	10,117	9,474	11,833	-	-

The data presented in Table 6 highlight the distinct cross-cultural differences between Chinese scholars in humanities and social sciences and native English speakers when it comes to the usage of lesser relevance markers. Out of the five major pragmatic functions, Chinese scholars use three categories less frequently, namely, Informing Evaluation, Topic Handling, and Interactivity. Particularly, the pragmatic function of Interactivity is significantly underutilized by Chinese scholars, as evidenced by a statistically significant difference compared to native English speakers. This information provides valuable implications regarding the communication and language patterns in these academic fields, shedding light on the need for further research and understanding of cross-cultural discourse.

In this paragraph, further normalization and chi-square paired testing were performed on two datasets, namely, CASEC-HS and MICASE-HS, to identify specific metalinguistic nouns of lesser relevance markers that show significant differences in their distribution in humanities and social sciences.

The results are presented in Table 7. The analysis of the table reveals five metalinguistic nouns with pragmatic functions that exhibit significant differences. Notably, the marker “about/with” (*χ*^2^ = −82.81, *p* = 0.000) is observed to be the least utilized lesser relevance marker by Chinese scholars in humanities and social sciences, while the marker “brief(ly)” (*χ*^2^ = 59.17, *p* = 0.000) is found to be the most frequently used. To gain a deeper understanding of these distinctions, a pragmatic function analysis will be conducted, focusing on Informing Evaluation and Topic Handling, as Interactivity only contains the marker “about/with” in both CASEC-T and CASEC-HS, indicating a similar explanation.

**Table 7:** Metalinguistic nouns of lesser relevance markers with significant differences in CASEC-HS and MICASE-HS.

Pragmatic functions of lesser relevance markers with significant difference		CASEC-HS frequency	MICASE-HS frequency	CASEC-HS normalized frequency	MICASE-HS normalized frequency	Chi-square value (*χ*^2^)	Significance level (*p*)
*Metalinguistic noun of informing evaluation*							
1	Anyway	28	154	92	180	−10.68	0.0011
2	By the way	4	76	13	89	−17.68	0.0000
3	Aside	2	26	7	30	−4.37	0.0366
*Metalinguistic noun of topic handling*							
3	A little bit	63	340	206	398	−23.23	0.0000
4	Brief(ly)	62	41	203	48	59.17	0.0000
*Metalinguistic noun of interactivity*							
5	About/with	2,593	8,880	8,486	10,386	−82.81	0.0000

#### 5.3.1 Pragmatic functional analysis of informing evaluation

Table 7 indicates that Chinese scholars in humanities and social sciences differ significantly from English native speakers in the use of three lesser relevance markers for informing evaluation, namely, “anyway,” “by the way,” and “aside.” Let us take two examples.

In English native contexts, the lesser relevance marker “anyway” is often used to indicate a topic transition, especially from secondary information to primary information. English native speakers skillfully use “anyway” to adjust the context, enhance lecture fluency, and emphasize core viewpoints. For instance, after explaining a complex theory, a professor might say, “Anyway, the key point here is....” In this example, “anyway” acts as a turning point, helping the audience shift focus from complex theoretical details to key points. English native speakers tend to rely on such strategies to balance the depth and breadth of lectures and maintain audience attention.

In comparison, when Chinese scholars use “anyway,” they tend to use it more as a marker of topic transition without emphasizing core information.

(1) Someone put the video on TV, and that LED to a great, very large wire in Los Angeles. But anyway, so here, do not rob me means do not treat me like rotten king (HS-023).

In Example 1, the Chinese scholar uses “anyway” to signal a shift in topics from the previous scene description to the subsequent humorous expression. “Anyway” organizes information by separating the less relevant scene description from the core lecture content. This helps the audience grasp the logical progression of information by clearly distinguishing key and secondary topics. Additionally, it adds a touch of humor and relaxation to the formal academic lecture atmosphere. By employing this strategy, key information is conveyed while creating a more engaging lecture environment through appropriate humor, enhancing audience attention and interest in learning (Abdi, 2002; Abdollahzadeh, 2011). However, such usage of “anyway” is relatively less common among Chinese scholars, possibly due to the traditional academic lecture atmosphere and cultural background in China. Many Chinese scholars tend to maintain the formality and rigor of lectures, avoiding the insertion of too many relaxed or humorous elements.

In English native contexts, “by the way” is often used as a tool to introduce additional information. Speakers use this expression when they want to insert relevant but non-critical information between main topics. This not only deepens the content of the lecture but also enhances audience interest. For example, when discussing the results of a scientific experiment, a professor might say, “By the way, this method was first developed by....” Here, “by the way” introduces information related to the topic but not crucial to it, enriching the lecture content.

Unlike English native speakers, Chinese scholars may be more cautious in using “by the way.”

(2) By the way the uh direct purchases actually reported in the multimedia data mining workshop if you are interested you can actually uh get the uh paper from the uh from the proceedings (HS-067).

In Example 2, the Chinese scholar uses “by the way” to supplement the preceding content by introducing new relevant information, similar to adding an extra annotation or footnote. This use of “by the way” inserts previously omitted but relevant supplementary information between the main contents of the lecture, making the lecture content richer and more comprehensive and presenting information holistically. Furthermore, this strategy enhances the connection between lecture topics, making it easier for the audience to grasp key points (Ädel, 2010; Björkman, 2011). However, such a strategy is relatively rare in Chinese scholars’ lecture expressions, possibly due to their tendency to maintain coherence and rigor in lectures. They tend to avoid expressions that may make the lecture appear irrelevant or unrigorous. Therefore, compared to English native speakers, Chinese scholars may be more conservative in their use of “by the way” as a lesser relevance marker.

In summary, Chinese scholars in humanities and social sciences deviate from English native speakers in their use of certain lesser relevance markers, such as “anyway” and “by the way.” These markers serve different purposes and have different implications in the two cultures. In English, “anyway” is frequently used to signal a transition in topics and emphasize core information. It helps maintain audience attention and lecture fluency. On the other hand, Chinese scholars tend to use “anyway” more as a marker of topic transition without emphasizing core information. It helps organize information and adds humor to the lecture atmosphere, but it is less commonly used due to the formal academic lecture atmosphere in China. Similarly, in English, “by the way” is used to introduce additional but non-critical information, enhancing the lecture content. Chinese scholars, however, are more cautious in using “by the way” and tend to maintain coherence and rigor in their lectures. These distinctions highlight the differences between pragmatic cultures and academic traditions. To improve lecture communication effects, Chinese scholars can learn from international academic conventions and strike a balance between rigor and vibrancy in their lectures.

#### 5.3.2 Pragmatic functional analysis of topic handling

Table 7 demonstrates significant differences between Chinese scholars in humanities and social sciences and English native speakers in their use of lesser relevance markers for topic handling. English native speakers employ the phrase “a little bit” primarily to diminish the importance of the topic, creating a more relaxed and less weighty tone. This prepares the audience for a relatively simple explanation and allows them to receive information in a relaxed manner, without feeling pressured. Such flexible and informal usage reflects the communicative norms of Western culture (Crismore et al., 1993; Fuertes-Olivera et al., 2001). In contrast, Chinese scholars use “a little bit” less frequently and prefer precise and accurate words to convey their thoughts. This preference may stem from the Chinese tradition of expression, which emphasizes precision and standardization. Additionally, the less frequent use of “a little bit” may be attributed to Chinese scholars’ non-native English background, suggesting a search for more specific expressions.

1. And finally, I want to talk a little bit about collaboration and project management (HS-033).

Example 3 illustrates how a Chinese scholar uses the phrase “a little bit” to indicate that the subsequent content is of minor importance and can be understood with minimal effort. This usage allows the scholar to organize the content by topic and helps the audience distinguish between main and secondary topics, enabling them to focus on the core content. However, this strategy is not commonly employed in Chinese scholars’ lectures.

On the other hand, English native speakers use the word “brief” to signify that they will provide a concise and fast explanation of a topic. This usage serves as a guide for the audience, indicating that they need not invest excessive energy in that particular section as it serves as an introduction or background for more important topics. This approach aligns with a common strategy in Western academic lectures, which involves setting clear structures to direct the audience’s attention (Harwood, 2005; Jalilifar and Alipour, 2014). In contrast, Chinese scholars use “brief” more frequently, possibly due to its widespread usage in academic settings. This reliance on fixed expressions in English may be related to the characteristics of English education in China, which stresses standardized and formulaic expressions.

1. So let me first give you a very brief introduction to this problem for taxonomy, construction, we have the input as text corpus (HS-054).

Example 4 showcases how a Chinese scholar employs a “brief introduction” to signal that the following explanation of a problem will be concise. This usage aids in content organization by clearly defining the length of a particular section, allowing the audience to process it in less time. Consequently, this strategy enables the lecture to cover more research content within a limited time. However, this use of “brief” is more prevalent in Chinese scholars’ lectures.

In summary, Chinese scholars in humanities and social sciences differ from English native speakers in their use of lesser relevance markers for topic handling. English native speakers use phrases such as “a little bit” to downplay the importance of a topic and create a more relaxed tone. This allows the audience to receive information without feeling pressured. In contrast, Chinese scholars prefer precise and accurate words and use “a little bit” less frequently. This may be due to the emphasis on precision and standardization in Chinese expression. Additionally, the less frequent use of “a little bit” may be attributed to Chinese scholars’ non-native English background. Chinese scholars also rely more on the word “brief” to indicate a concise explanation of a topic. This aligns with the strategy of setting clear structures in Western academic lectures. However, Chinese scholars’ use of “brief” may be influenced by the characteristics of English education in China, which emphasizes standardized expressions. Overall, Chinese scholars should adjust their strategies and acquire effective interactive expressions to enhance the interactive effect of their academic lectures in international contexts.

### 5.4 Cross-disciplinary analysis of pragmatic functions of lesser relevance markers within Chinese science and engineering scholars and humanities and social sciences scholars

Based on the aforementioned analysis, it is evident that Chinese scholars demonstrate significant differences in their use of lesser relevance markers for the five major pragmatic functions when compared to English native speakers. These variations can largely be attributed to cultural differences. To further investigate the impact of disciplinary variance within the group of Chinese scholars, an examination of their use of lesser relevance markers in academic spoken English is conducted. By distinguishing Chinese Scholars (CASEC) into science and engineering scholars (CASEC-T) and humanities and social sciences scholars (CASEC-HS) and employing the five major classifications of pragmatic functions, the outcomes are presented in Table 8.

**Table 8:** Metalinguistic nouns of lesser relevance markers with significant differences between CASEC-T and MICASE-HS.

Pragmatic functions of lesser relevance markers with Significant Difference		CASEC-HS frequency	MICASE-HS frequency	CASEC-HS normalized frequency	MICASE-HS normalized frequency	Chi-square value (*χ*^2^)	Significance level (*p*)
*Metalinguistic noun of informing evaluation*							
1	Anyway	11	28	21	92	−19.53	0.0000
*Metalinguistic noun of topic handling*							
2	A little bit	53	63	100	206	−15.20	0.0001
3	Brief(ly)	72	62	135	203	−5.16	0.0232
*Metalinguistic noun of appraisal*							
4	Not know	3	9	6	29	−6.12	0.0134
*Metalinguistic noun of interactivity*							
5	About/with	3,815	2,593	7,163	8,486	−44.63	0.0000

According to Table 8, overall, Chinese science and engineering scholars and humanities and social sciences scholars show little significant difference in the specific metalinguistic nouns of lesser relevance markers in academic spoken English. Only five markers show significant differences, but these differences exist across four functions.

First, there is a significant difference in using the marker “anyway” in Informing Evaluation. Specific examples are provided below:

1. Well, the train is passing in Sims. Ok anyway, so you feel trading the stocks before you should be familiar with all these indicators unless you traded stocks blindly hopefully not (T-035).
2. And anyway, the civil society in China eh have has 2 different meanings, comparing the eh official usage and the academic usage (HS-002).

In Example 5, the Chinese science and engineering scholar uses “anyway” to achieve topic transition, leading to the subsequent analogy section. In Example 6, the Chinese humanities and social sciences scholar uses “anyway” to supplement the preceding content, serving an explanatory function. However, after chi-square paired testing, it is found that compared with Chinese humanities and social sciences scholars, Chinese science and engineering scholars rarely use “anyway” for informing evaluation (*χ*^2^ = −19.53, *p* = 0.000). This phenomenon may stem from the technical nature of science and engineering academic lectures (Hempel and Degand, 2008; Khedri et al., 2013). In comparison with humanities and social sciences, science and engineering academic lectures place greater emphasis on rigor and logic. Therefore, the use of “anyway” for informing evaluation is relatively low in the expression of science and engineering scholars.

Overall, it is clear that Chinese scholars differ significantly from English native speakers in their use of lesser relevance markers for pragmatic functions. These differences are largely due to cultural disparities. Upon examining the usage of lesser relevance markers in academic spoken English by Chinese Scholars (CASEC), the results show minimal differences between science and engineering scholars (CASEC-T) and humanities and social sciences scholars (CASEC-HS). Only five markers exhibit significant differences, which are spread across four functions. However, there is a notable discrepancy in the use of the marker “anyway” in the function of Informing Evaluation. For instance, Chinese science and engineering scholars rarely employ “anyway” for informing evaluation, unlike their humanities and social sciences counterparts. This discrepancy can be attributed to the technical nature of science and engineering academic lectures, which prioritize rigor and logic rather than expressive evaluation.

Second, there are significant differences in using the markers “a little bit” and “brief(ly)” in Topic Handling. Specific examples of “a little bit” are as follows:

1. Our first start with background. I would tell a little bit about our previous work on retrieval models based on past constraint random walks (T-067).
2. And finally, I want to talk a little bit about collaboration and project management (China Ready – Mandarin Chinese for Hospitality and Tourism, HS-002).

In Examples 7 and 8, Chinese scholars use the phrase “a little bit” to indicate that the following content can be understood quickly. However, this usage is much less common among Chinese science and engineering scholars compared to Chinese humanities and social sciences scholars (*χ*^2^ = −15.20, *p* = 0.000). This difference is also evident in the use of “brief(ly)” (*χ*^2^ = −5.155, *p* = 0.023).

It is clear that Chinese scholars often use the phrase “a little bit” to indicate that the following content can be understood quickly. However, this usage is much less common among Chinese science and engineering scholars compared to their counterparts in humanities and social sciences. This preference for specific and detailed expressions in academic lectures among science and engineering scholars may be attributed to the technical and professional nature of their fields. In contrast, humanities and social sciences scholars prioritize more general and concise expressions. These cross-cultural distinctions in the use of language have important implications for communication and understanding between different disciplinary fields.

Third, there is no significant difference in using the markers of the Lecturer Strategy. This may be attributed to the formal nature of traditional Chinese culture that requires serious expression on formal occasions.

Fourth, there is a significant difference in using the marker “not know” of Appraisal. Specific examples are provided below:

1. We can decompose the drug distribution into some local factors and also use message propagation to estimate posterior distribution of Y given X and given parameters. And for those who do not know message propagation (T-089).
2. This is. You see, in this case, you probably do not know why, okay? You do not know why (HS-049).

In Examples 9 and 10, Chinese scholars use the phrase “not know” to imply the lack of knowledge or understanding on the part of a third party. This reflects the implicitness in expressions commonly used by Chinese scholars. However, there are fewer occurrences of “not know” among Chinese science and engineering scholars compared to Chinese humanities and social sciences scholars (*χ*^2^ = −6.122, *p* = 0.013).

This indicates that Chinese science and engineering scholars tend to prefer indirect ways of assigning tasks or avoiding appraisal, whereas Chinese humanities and social sciences scholars use a more implicit approach. This difference may arise from the different characteristics and requirements of the disciplinary fields. In science and engineering, scholars prioritize the direct expression of viewpoints (Salager-Meyer, 1994; Zare and Tavakoli, 2016), while humanities and social sciences scholars value discussion and exchange. Therefore, science and engineering scholars use indirect expressions to ensure accurate information delivery while showing respect for others.

Finally, in terms of Interactivity in academic spoken English, the study finds that there is a notable difference in the usage of the preposition “about/with” between Chinese science and engineering scholars and humanities and social sciences scholars. Chinese scholars, in general, tend to use “about/with” less frequently, with science and engineering scholars using it significantly less than humanities and social sciences scholars (*χ*^2^ = −44.63, *p* = 0.000). This difference can be attributed to the more tight and logical lecture content in science and engineering, which leads to distinct linguistic patterns. These findings highlight an important cross-cultural distinction in academic spoken English and have implications for understanding and improving intercultural communication.

## 6 Conclusion

This study investigates cross-cultural differences in the usage of lesser relevance markers in the academic spoken English of Chinese scholars in humanities and social sciences. The data presented in Tables 6, 7 reveal these differences across the five major pragmatic functions of lesser relevance markers. Chinese scholars use categories such as Informing Evaluation, Topic Handling, and Interactivity less frequently compared to native English speakers. Specifically, the function of Interactivity is significantly underutilized by Chinese scholars. The study also explores specific metalinguistic nouns of lesser relevance markers that show significant differences, providing examples and explanations of their usage by Chinese scholars. Furthermore, the analysis highlights differences in the use of these markers between science and engineering scholars and humanities and social sciences scholars. Chinese science and engineering scholars use markers such as “anyway” and “a little bit” less frequently compared to their counterparts in humanities and social sciences. Additionally, the study illustrates differences in the use of the marker “not know” in Appraisal and the preposition “about/with” in Interactivity between the two groups of scholars. These cross-disciplinary distinctions underscore the influence of disciplinary variance on the usage of lesser relevance markers, which can be attributed to cultural and disciplinary factors. Particularly, Chinese scholars tend to avoid using humor and instead prefer fixed expressions, reflecting cultural norms and the process of English language acquisition. Furthermore, science and engineering scholars are less inclined to use topic transition markers, indicating a greater emphasis on lecture rigor. It is recommended that all scholars increase their use of lesser relevance markers to enhance communication. In terms of disciplinary differences, science and engineering scholars use lesser relevance markers less frequently than humanities and social sciences scholars, suggesting more condensed lectures. Conversely, humanities scholars tend to provide more explicit instructions to the audience, reflecting pragmatic norms. The findings of this study will facilitate cross-cultural and cross-disciplinary communication and provide insights for teaching reform. However, it is important to acknowledge the study’s limitations, including the lack of analysis based on other dimensions such as discourse type and speaker identity. Therefore, further multidimensional research is necessary to gain a comprehensive understanding across different contexts. This study offers valuable insights for the development of academic communication and the teaching of spoken English. Educators can utilize this research to formulate more effective policies and curricula to meet the challenges of globalization in academia. Moreover, this research has a profound influence and value in teaching reform as it not only improves teaching quality but also lays a solid foundation for cultivating talents with an international perspective and cross-cultural communication skills. Additionally, by updating textbooks, enriching teaching methods, enhancing teacher training, and promoting multicultural understanding, this research provides valuable resources and tools for educators and students. Finally, education authorities and schools can utilize the findings of this research to formulate more effective and forward-looking teaching policies that address the increasingly globalized academic environment and challenges.

## Data availability statement

The raw data supporting the conclusions of this article will be made available by the authors, without undue reservation.

## Author contributions

YY: Conceptualization, Data curation, Formal analysis, Investigation, Methodology, Project administration, Software, Validation, Writing – original draft, Writing – review & editing. GZ: Project administration, Supervision, Validation, Writing – review & editing.
